# Therapeutic Potential and Molecular Mechanisms of Echinacoside in Neurodegenerative Diseases

**DOI:** 10.3389/fphar.2022.841110

**Published:** 2022-02-04

**Authors:** Jin Li, Hongni Yu, Chuan Yang, Tao Ma, Yuan Dai

**Affiliations:** ^1^ School of Health Preservation and Rehabilitation, Chengdu University of Traditional Chinese Medicine, Chengdu, China; ^2^ Dongfang Hospital, Beijing University of Chinese Medicine, Beijing, China; ^3^ School of Pharmacy, Chengdu University of Traditional Chinese Medicine, Chengdu, China

**Keywords:** echinacoside, neurodegenerative diseases, Alzheimer’s disease, Parkinson’s disease, amyotrophic lateral sclerosis, vascular dementia

## Abstract

Echinacoside (ECH) is a natural phenylethanoid glycoside (PhG) in *Cistanche tubulosa*. A large number of studies have shown that ECH has very promising potential in the inhibition of neurodegenerative disease progression. Experimental studies strongly suggest that ECH exhibits a variety of beneficial effects associated with in neuronal function, including protecting mitochondrial function, anti-oxidative stress, anti-inflammatory, anti-endoplasmic reticulum stress (ERS), regulating autophagy and so on. The aim of this paper is to provide an extensive and actual summarization of ECH and its neuroprotective efficacy in prevention and treatment of neurodegenerative diseases, including Alzheimer’s disease (AD), Parkinson’s disease (PD), amyotrophic lateral sclerosis (ALS), and so on, based on published data from both *in vivo* and *in vitro* studies. There is a growing evidence that ECH may serve as an efficacious and safe substance in the future to counteract neurodegenerative disease.

## 1 Introduction

With the increase in population aging, neurodegenerative diseases are the most prevalent and fastest growing disorders of the elderly worldwide, which are endangering human health and cause a heavy financial burden on society (Erkkinen et al., 2018). The number of the dementia elderly is expected to increase to 130 million by 2050, and the annual socio-economic cost per patient is US$19,144.36, the global cost is estimated to reach US$9.12 trillion by 2050 (Jia et al., 2018; Hansson, 2021). Neurodegenerative diseases mainly include Alzheimer’s disease (AD), Parkinson’s disease (PD), Huntington’s disease (HD), amyotrophic lateral sclerosis (ALS), multiple sclerosis (MS), vascular dementia (VD), and so on (Gitler et al., 2017). Neuronal damage, mitochondrial dysfunction, oxidative stress and neuroinflammation are their common pathogenesis feature. The neurodegenerative diseases are mainly manifested as memory and cognitive impairment, and abnormal movement in clinic (Enogieru et al., 2018). Up to now, the therapeutic strategy for neurodegenerative diseases mainly focuses on improving symptoms. Therefore, it is urgent to explore and develop novel drugs with therapeutic potential directed at the pathogenesis of neurodegenerative disease.

**FIGURE 1:**
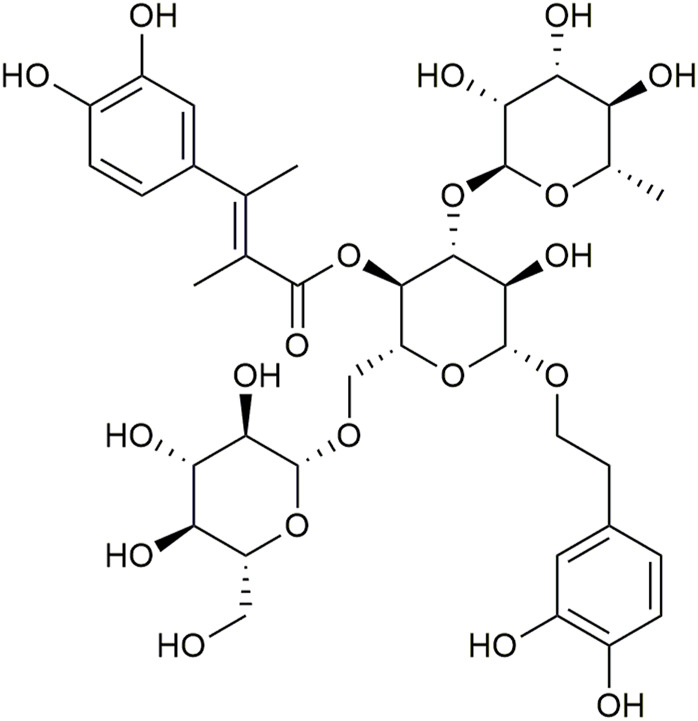
Chemical structure of Echinacoside (ECH).

Echinacoside (ECH, Figure 1) is a phenylethanoid glycoside (PhG), and was first extracted from the rhizome of *Echinacea angustifolia* DC. It is not only one of the key effective components of *Echinacea* (Jiang and Tu, 2009; Zhu et al., 2013; Li et al., 2018), but also abundant in other natural plants, such as *Scrophulariae Radix*, *Rehmanniae Radix*, *Cistanches Herba*, etc. Among them, *Cistanche tubulosa* contains the highest content of ECH reaching about 30% (w/w) (Tian XY et al., 2021). The molecular formula of ECH is C_35_H_46_O_20_ (Figure 1) and its chemical structure is composed of a sugar group, a phenylpropenyl group, and a phenylethanol group (Wu et al., 2020). Recently, ECH has shown a variety of important pharmacological activities, such as anti-inflammatory, protection of mitochondrial function, anti-oxidation, anti-neurotoxicity, anti-endoplasmic reticulum stress (ERS), anti-apoptosis, and neuroprotective effect (Wang et al., 2015; Chen et al., 2018; Ma et al., 2019; Wei et al., 2019; Wu et al., 2020). In addition, the metabolites of ECH C6-C3 and C6-C2 may have certain neuroprotective activities, which can directly supplement the neurotransmitter defects (Song et al., 2019). This review presented current and innovative results concerning the pharmacology and the efficacy of ECH in the treatment of neurodegenerative diseases, focusing on its mechanism of action in AD, PD, VD, and ALS, etc.

## 2 Alzheimer’s Disease

AD is an irreversible and progressive chronic central neurodegenerative disease. It is the most common type of dementia and is becoming a major challenge to global health and social care (Alzheimer’s Association, 2020). Patients with AD usually suffer from significant memory impairment, language difficulties, decreased executive and visuospatial functions, and other degrees of cognitive impairment, as well as personality and behavior changes (Hansson, 2021). The pathogenesis mainly includes oxidative stress, mitochondrial abnormalities, neuroinflammation, abnormal accumulation of extracellular Amyloid-β (Aβ) plaques, and so on (Lane et al., 2018; Magalingam et al., 2018). AD involves multiple pathogeneses. The current treatment strategy for a single pathway has been proved to be insufficient. Recent evidences indicate that ECH has very extensive neuropharmacological activities. It may be a potential natural active ingredient with broad-spectrum and multiple target effects in the treatment of AD.

### 2.1 The Production and Toxicity of Amyloid-β

Excessive accumulation of Aβ oligomers and soluble aggregates outside the central nerve cells of the brain is one of the important causes of AD (Imbimbo and Watling, 2019). Beta site amyloid precursor protein cleaving enzyme 1 (BACE1) is the key rate-limiting enzyme for amyloid precursor protein (APP) processing to produce Aβ, which can catalyze the initial cleavage of APP and produce Aβ. Dai et al. confirm that ECH affects Protein kinase-like endoplasmic reticulum kinase (PERK), inhibits the PERK/eIF2α pathway to reduce ERS, and regulates F-actin remodeling to reduce the excessive accumulation of Aβ and the expression of BACE1 protein by using immunohistochemistry, Aβ plaque load quantification, Aβ ELISA, RNA isolation, quantitative PCR, Western blot analysis, BACE1 activity assay and Transmission Electron Microscopy in APPswe/PS1dE9 (2 × Tg-AD) mice (Dai et al., 2020). In the hen egg-white lysozyme (HEWL) model system, Zhang et al. used spectroscopic analyses, electron microscopy, cell viability assay, and hemolysis assay to find that ECH can inhibit the conversion of HEWL in a dose-dependent manner, antagonize amyloidosis, destroy the structure of fibrils, and convert amyloid fibrils into amorphous aggregates (Zhang et al., 2015). In addition, in Amyloid β peptide 1-42 (Aβ 1-42)-treated SH-SY5Y cells, Shiao et al. found that ECH inhibited Aβ_1-42_ oligomerization, restored the cell viability that was reduced by Aβ_1-42_, and reduced acetylcholinesterase activity, which in turn reverses cortical cholinergic dysfunction caused by Aβ_1-42_ in Aβ_1-42_-infused rat (Shiao et al., 2017). Wu et al. used the AD rats induced by injecting Aβ_1-42_ and found ECH can ameliorate the cognitive deficits, decrease amyloid deposition and reverse cholinergic and hippocampal dopaminergic dysfunction caused by Aβ_1-42_ (Wu et al., 2014).

### 2.2 Oxidative Stress

The central nervous system requires high energy, so it is one of the organs that is vulnerable to hypoxia (Wilson et al., 2009). The presence of oxidative damage in neuronal lipids and proteins is an important feature of AD. The binding of redox-active metal ions to Aβ and mitochondrial dysfunction can catalyze the production of reactive oxygen species (ROS) (Cheignon et al., 2018). Studies have shown that ECH has significant antioxidant and free radical scavenging properties. Nuclear factor-erythroid 2-related factor 2 (Nrf2) is one of the most important transcription factors in oxidative stress. ECH significantly decreased the Kelch-like ECH-associated protein-1 (Keap1) protein expression along with the substantial nuclear accumulation of Nrf2 in hippocampus. ECH further elevated the expression of heme oxygenase-1 (HO-1), NAD(P)H quinone oxidoreductase 1 (NQO1) and γ-glutamyl cysteine Synthetase (γ-GCS). Then, Nrf2 promotes the transcription of key antioxidative enzymes, such as superoxide dismutase (SOD) and phase II detoxifying genes (such as NQO1), and regulates the Keap1-Nrf2-ARE pathway to depress oxidative stress and mitochondrial dysfunction (Zheng et al., 2019). In the *Caenorhabditis elegans*, ECH suppressed oxidative stress via the DR pathway and the insulin/IGF signaling (IIS) pathway, which triggers the nuclear localization of DAF-16. Then DAF-16 regulates target genes which participate in lifespan regulation and stress resistance. ECH also increased its two downstream targets, namely superoxide dismutase (sod-3) and small heat shock protein 16.2 (hsp-16.2) which involved in oxidative damage (Mukhopadhyay et al., 2006; Chen et al., 2018). Furthermore, ECH could ameliorate the pathology of AD through decreasing the formation of ROS and the accumulation of intracellular free Ca^2+^, and improving the mitochondrial membrane integrity (Kuang et al., 2009).

### 2.3 Neuroprotection

Glutamate is the main excitatory neurotransmitter. Excessive release of glutamate and excessive excitement of N-methyl-D-aspartate receptor (NMDAR) can cause depolarization of nerve cell membranes and cause a large amount of Ca^2+^ influx, which is an important cause of neuronal degeneration and death (Cobley et al., 2018). According to the research results of Lu and others, in the rat cerebral cortex, ECH can reduce voltage-dependent Ca^2+^ influx and inhibit protein kinase C activity (Lu et al., 2016). Shiao et al. infuse Aβ_1-42_ into the brain cistern by an osmotic pump and found that ECH can inhibit AChE activity, reverse cortical cholinergic neuron dysfunction caused by Aβ deposition, and improve cognitive dysfunction caused by Aβ_1-42_. Achieve protection of nerves from toxic effects (Shiao et al., 2017).

### 2.4 Apoptosis

ECH can inhibit the release of cytochrome c (Cyt c) and the activation of caspase-3 through the extracellular signal-regulated kinase (ERK) pathway *in vitro* (Zhu et al., 2013). The members of the Bcl-2 family-like Bax and Bcl-2 have participated in apoptosis induced by the accumulation of ROS through the mitochondrial apoptotic pathway (Deng et al., 2004; Soane et al., 2008). ECH prevents a H_2_O_2_-induced increase of the Bax/Bcl-2 ratio to depress apoptosis in rat pheochromocytoma cell line (PC12 cells) (Kuang et al., 2009).

## 3 Parkinson’s Disease

PD is a chronic progressive neurodegenerative disease. Its clinical symptoms mainly include resting tremor, bradykinesia, muscle rigidity, and postural and gait disorders. The typical neuropathological features of PD are the degeneration of dopaminergic neurons located in the substantial nigra pars compacta (SNpc) of the midbrain and the formation of Lewy bodies, and α-synuclein and ubiquitin are the main components of Lewy bodies (Dunnett and Björklund, 1999). Recent studies have shown that the loss of dopaminergic terminals in the striatum, rather than the loss of SNpc neurons, is crucial for the occurrence of motor symptoms. With the aging of the global population, the prevalence of PD is expected to double in the next 20 years (Simon et al., 2020).

### 3.1 Neuroinflammation

ECH inhibits the activation of microglia and astrocytes in 6-hydroxydopamine (6-OHDA) subacute PD model mice and promotes the nerves of dopamine (DA), 3,4-dihydroxyphenylacetic acid (DOPAC), high vanillic acid (HVA), norepinephrine (NE), and serotonin (5-HT) in the striatum and extracellular fluid of the hippocampus. Secretion of nutritional factors (Chen et al., 2007), and finally, the apoptosis of DA neurons is reduced, and the pathological state of PD is improved. It is also reported in the literature that ECH can prevent the level of DA, DOPAC, and HVA in the right striatum of 1-methyl-4-phenyl-1,2,3,6-tetrahydropyridine (MPTP) model mice from decreasing (Zhang et al., 2021a). At the same time, inhibiting the reduction of striatal fibers, DA and DA transporters can improve gait disorders. *In vitro* experiments show that ECH can improve 6-OHDA-induced PC12 cell model cell viability, significantly enhance redox activity and mitochondrial membrane potential, reduce ROS production, and inhibit mitochondrial-mediated apoptosis (Chen et al., 2019). MPTP is converted into 1-methyl-4-phenylpyridinium (MPP^+^) by monoamine oxidase B in glial cell species. MPP^+^ produces neurotoxicity by generating ROS in DA neuron mitochondria (Ahmed et al., 2017). MPP^+^ is now widely used to induce damage in SH-SY5Y cell line to mimic the pathogenesis of PD. After ECH administration, it has been shown to attenuate DA neuron damages. Zhang et al. used proteomics to detect pro-inflammatory cytokines and found that seven cytokines including c5/c5a, interleukin-1beta (IL-1β), tumor necrosis factor-alpha (TNF-α), interleukin-2 (IL-2), and interleukin-4 (IL-4) were down-regulated by down-regulating p38 mitogen Pro-activated protein kinase (p38MAPK) and nuclear factor-kappa B (NF-κB) p52 (Zhang J et al., 2017; Liang et al., 2019) or regulate the activation of ROS/ATF3/CHOP pathway participate in the inhibition of inflammation in dopaminergic neurons in the midbrain the occurrence of the reaction, thereby inhibiting the occurrence of apoptosis, has a neuroprotective effect (Zhao et al., 2016). The excessive activation of microglia is closely related to neurotoxicity and participates in the main pathological development of PD. The inflammatory response mediated by activated microglia is the main component of the pathological process of PD. In the ECH group of MPTP model mice, the specific marker Iba-1 of microglia in the midbrain decreased, and ECH treatment inhibited the small activation of glial cells which improves inflammation in the brain (Ho, 2019). There are also reports in the literature that ECH improves the neuropathological state of PD mice through neuroprotective cell survival and inhibiting activated microglia-mediated NLRP3/CASP-1/IL-1β inflammation signals (Gao et al., 2020). In summary, ECH can depress the neuroinflammation that involved in the pathological progress of PD by multiple ways.

### 3.2 Apoptosis

Bcl-2 is the coding product of the Bcl-2 proto-oncogene and is an apoptosis-inhibiting protein. It promotes cell survival by inhibiting the permeability of the outer mitochondrial membrane, regulates the release of mitochondrial apoptotic factors, participates in the regulation of apoptosis, and promotes cells’ survive (Liu et al., 2019). In MPTP subacute PD model mice, ECH inhibits the release of mitochondrial Cyt c and caspase-8 and the lysis of caspase-3 by reducing the ratio of Bax/Bcl-2 in dopamine neurons in the substantia nigra and plays a role of inhibiting apoptosis.

### 3.3 Autophagy

In the pathological process of PD, the disorder of autophagy regulation will eventually lead to the accumulation of misfolded proteins and the damage of organelles. The autophagy-lysosome pathway can not only degrade proteins that cannot be degraded by the ubiquitin-proteasome pathway, but also degrade α-synuclein (Webb et al., 2003). Autophagy is the main pathway for the degradation of intracellular aggregates, and mechanistic target of rapamycin (mTOR) kinase is the key regulatory site for autophagy (Glick et al., 2010). By regulating the autophagy-lysosome pathway and increasing the degradation of autophagy and α-synuclein, the addition of ECH has significant advantages in improving the clinical efficacy and clinical symptoms of PD. Zhang et al. used MPTP to create a subacute PD mouse model. ECH can significantly improve the neurobehavior of PD mice by upregulating the survival signal p-AKT/AKT The expression of mTOR inhibits the expression of mTOR, thereby promoting the clearance of α-synuclein and the degradation of the autophagy substrate P62, exerting a neuroprotective effect (Zhang et al., 2021b). Sirtuins are nicotinamide adenine dinucleotide (NAD^+^)-dependent deacetylases that play an important role in neuronal development and aging, which also have neuroprotective effects in PD models *in vivo* and *in vitro* (Donmez and Outeiro, 2013). It has been proved that sirtuin 1 (SIRT1) promotes the transcription of HSP70 (heat shock protein) and other molecular chaperones by deacetylating heat shock factor 1, and is an effective regulator of autophagy. Experiments have shown that ECH binds to SIRT1, and the binding product activates forkhead box subgroup O1 (FoxO1) to cause autophagy gene transcription. And translation, promote the autophagic degradation of α-synuclein, which can reverse the damage of dopaminergic neurons (Chen et al., 2019).

### 3.4 Nourishment of Nerves

The neurotrophic factor is a protein for the growth and survival of neurons, including nerve growth factor (NGF), brain-derived neurotrophic factor (BDNF), and glial cell-derived neurotrophic factor (GDNF). It can promote the growth and development of nerves and axons. The increased incidence of neuronal apoptosis and the decreased protective effect of neurotrophic factors caused by various pathological factors are the basis of dopaminergic neuron degeneration (Barker et al., 2020). GDNF has the strongest neurotrophic factor that protects and promotes the repairment of dopaminergic neurons. ECH improved the viability of MPP^+^-treated cell *in vivo*, and increased the expression of tyrosine hydroxylase (TH), GDNF, GDNF family receptor α (GFRα1) and Ret in cells of substantia nigra (SN) of MPTP-induced PD mouse model (Xu et al., 2016).

### 3.5 Endoplasmic Reticulum Stress

Seipin/BSCL2 has been identified as the pathogenic gene for Berardinelli-Seip congenital lipodystrophy type 2 (BSCL2) and induces the most severe form of lipodystrophy, characterized by an almost complete absence of adipose tissue and associated metabolic disorders. Seipin is a global membrane protein of the endoplasmic reticulum (ER) that plays a key role in adipogenesis, lipid droplet homogenization, and cellular triglyceride lipolysis (Wang et al., 2018). Seipin accumulation strongly impaired adipocyte isotropy and leads to lipodystrophy, showing potential neural involvement. Chronic ERS leads to the accumulation of α-synuclein, and unfolded proteins in ER further leads to neuronal death (Licker et al., 2014). ECH attenuates the accumulation of Seipin (BSCL2) in 6-OHDA-induced rat models by promoting its ubiquitination and degradation, thereby reducing the activation of ERS related pathways (Zhang Y et al., 2017).

### 3.6 Mitochondrial Dysfunction

Mitochondria are key organelles for adenosine 5′-triphosphate (ATP) production through oxidative phosphorylation (OXPHOS), which is necessary for normal cellular functions. OXPHOS generates electron donor NADH and flavin adenine dinucleotide (FADH_2_) through electron transport chain (ETC) transmission to produce ATP (Filograna et al., 2021). The spare respiratory capacity (SRC) that mitochondria reserve under normal physiological conditions is critical for cell survival under stress when energy needs increase or oxygen depletion occurs. Complex I is the main entry point of ETC and is the site that mainly produces lesions or injuries. MMP induces the mouse model of PD by inhibiting complex I, suggesting that complex I may be the core of PD pathogenesis (Iverson, 2013), and it is another entry point for ETC and is used to reduce equivalents. There is evidence that complex II is a key regulator of neuroprotection, and complex II is a major source of SRC. In the human neuroblastoma SH-SY5Y cell line, ECH selectively attenuated cell damage and reversed complex I by increasing the activity of complex II to ameliorate the mitochondrial respiratory disorder and bioenergy shortage (Ma et al., 2019). ECH could also increase the mitochondrial membrane potential, and restore the mitochondrial energy supply in 6-OHDA-treated PC12 cells.

## 4 Other Neurodegenerative Disease

Compared with the study of the mechanism that ECH is used to treat AD and PD, the mechanism of ECH’s potential therapeutic effect on ALS, VD, and other diseases research is still very few. ALS is a type of motor neuron disease characterized by the degeneration of more than motor neurons and lower motor neurons. Yang Tian’s research team found that 10 μM ECH promoted the expression of glutamate transporter 1 (GLT1) in astrocytes, and found that ECH can significantly improve neuron survival and synapse loss treated with superoxide dismutase 1 (SOD1) astrocyte conditioned medium. Western blot and MTS were used. As well as immunohistochemistry and confocal imaging, this clarifies to a certain extent that ECH has a potential therapeutic effect on ALS (Tian Y et al., 2021). However, the molecular mechanism of 10 μM ECH promoting GLT1 expression needs more exploration. VD is a disease of severe cognitive impairment often caused by cerebrovascular diseases such as hemorrhagic or ischemic stroke. In previous studies, ECH has been found to have a certain promoting effect on the restoration of ACh and choline levels in the hippocampus and striatum of VD model rats, and it can also significantly increase the activity of AChE. The recovery of the cholinergic nervous system of VD rats has a promoting effect, but the specific molecular mechanism remains to be further discovered (Liu et al., 2013). And Xu Liu et al. also showed that ECH can up-regulate the expression of GDNF in the hippocampus by regulating the level of mitochondrial oxidation, thereby reducing the ischemic damage of VD rat neurons and improving learning and memory function (Liu et al., 2017).

## 5 Conclusion and Future Directions

**FIGURE 2:**
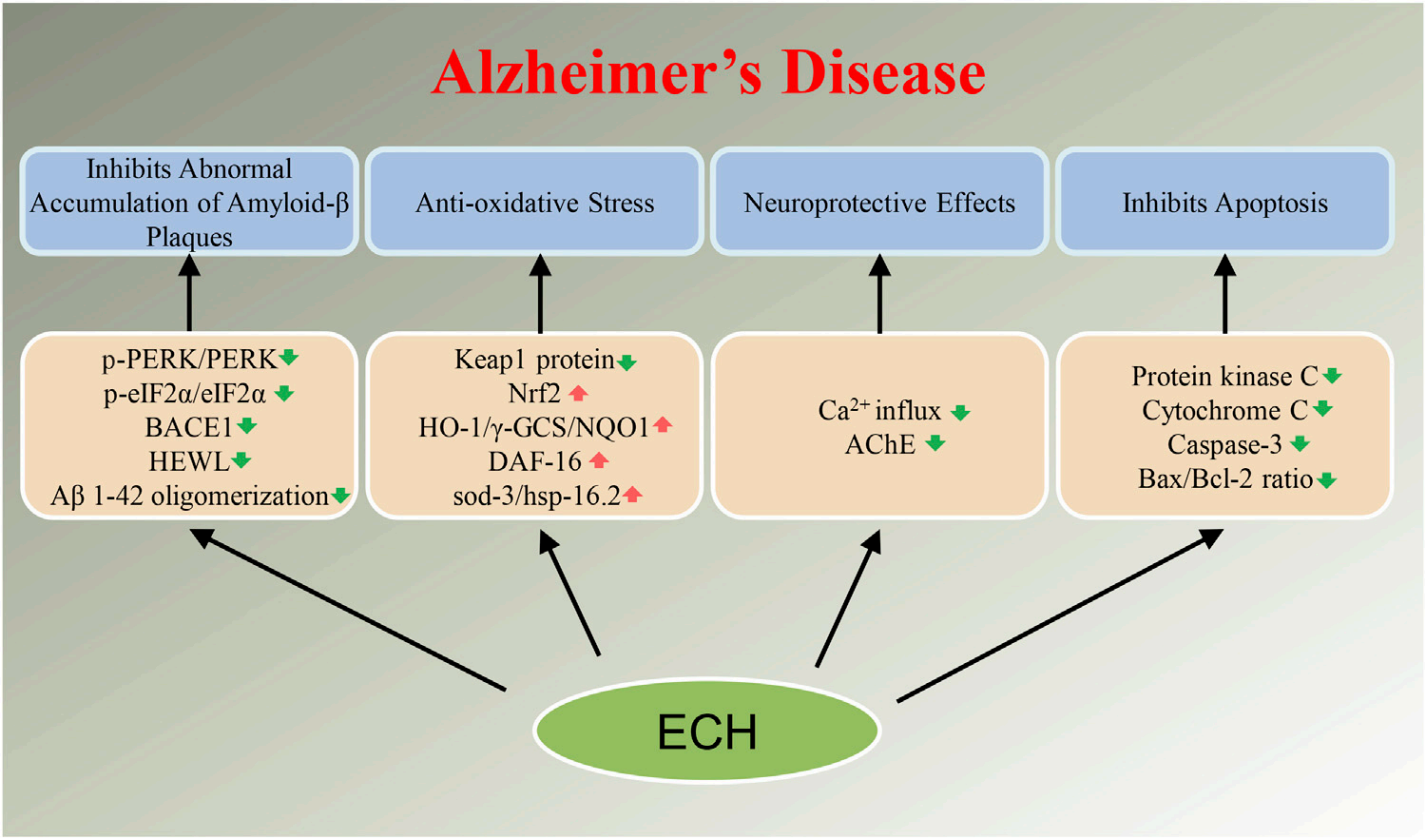
Diagram with neuroprotective mechanisms of Echinacoside (ECH) in Alzheimer’s disease (AD). ECH can improve neurodegenerative diseases by regulating target genes or target proteins on abnormal accumulation of Amyloid-β plaques, oxidative stress, apoptosis, and neurotoxicity signaling pathways.

**FIGURE 3:**
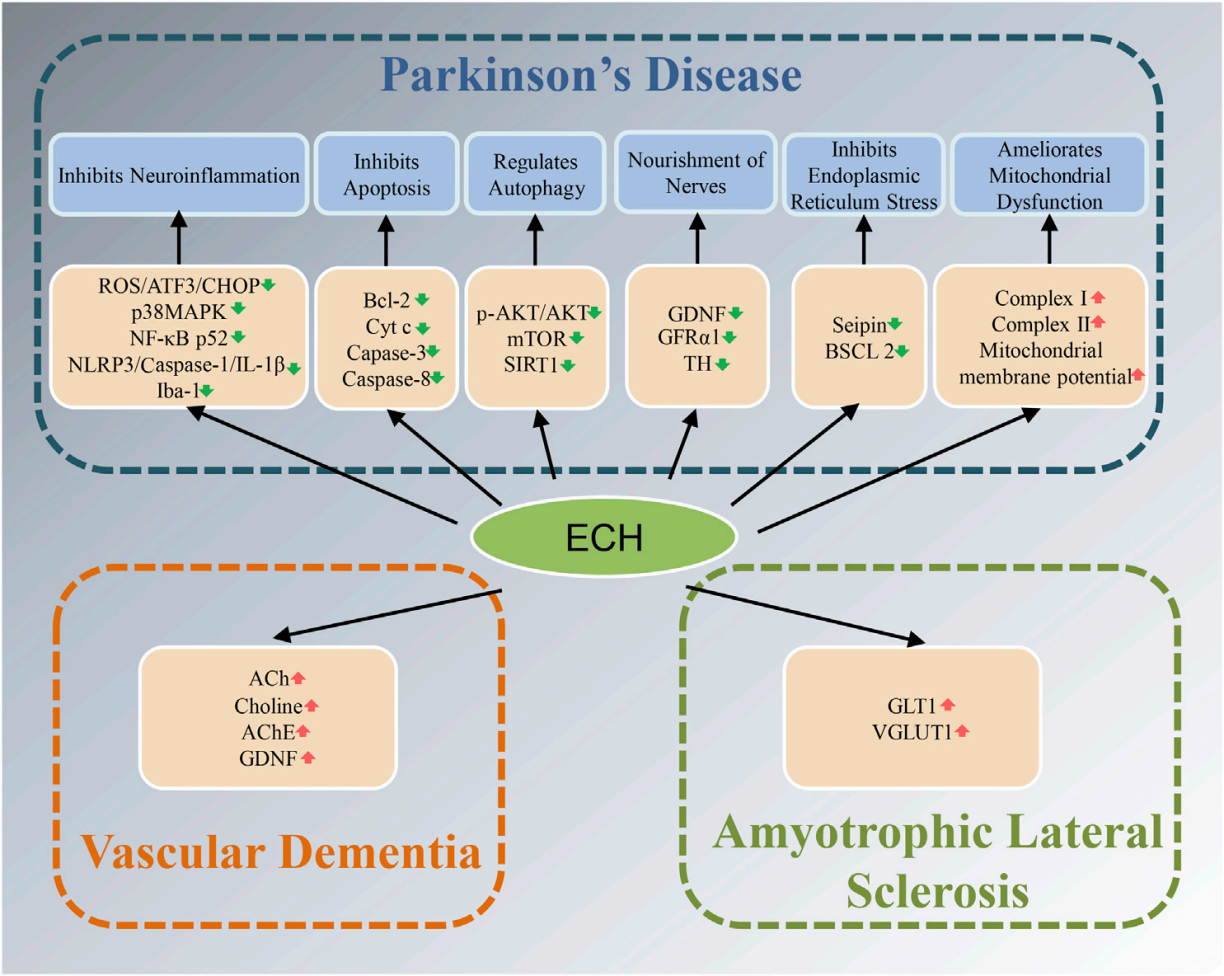
Diagram with neuroprotective mechanisms of Echinacoside (ECH) in Parkinson’s disease (PD), amyotrophic lateral sclerosis (ALS), and vascular dementia (VD). ECH can improve neurodegenerative diseases by improving oxidative stress, neuroinflammation, apoptosis, autophagy, nourishment of nerves, and mitochondrial dysfunction signaling pathways.

Neurodegenerative diseases have caused a tremendous burden to patient’s family and society worldwide. Up to now, therapeutic drugs remain very scarce. Natural active ingredients may be one of the promising drug discovery strategies for AD treatment. As a natural PhG, ECH has been confirmed to be have multiple neuroprotective effects such as anti-oxidative stress, anti-apoptosis, anti-neuroinflammation, inhibit the accumulation of toxic protein and regulate autophagy and ERS (Figure 2,3). All of this implied that ECH possess broad-spectrum and multiple target neuropharmacological effects, suggesting ECH may be a potential candidate compounds to develop therapeutic drug for treating neurodegenerative diseases with multi-target collaborative intervention. Here, we enumerate the neuroprotective effects of ECH against neurodegenerative diseases based on current reports and results (Table 1).

**TABLE 1:** Neuroprotective effects of Echinacoside against neurodegenerative diseases.

Disease	Model	Mechanism	Target protein	Result	References
AD	APPswe/PS1dE9 (2 × Tg-AD) mice	inhibit the abnormal accumulation of Aβ plaques	PERK/eIF2α	reduce ERS and regulate F-actin remodeling to reduce the excessive accumulation of Aβ and the expression of BACE1	Dai et al. (2020)
	Aβ_1-42_-injected AD rats	inhibit the abnormal accumulation of Aβ plaques	AChE	ameliorate the cognitive deficits, decrease Aβ deposition and reverse cholinergic and hippocampal dopaminergic dysfunction caused by Aβ_1-42_	Wu et al. (2014)
	Aβ_1-42_-treated SH-SY5Y cells	inhibit the abnormal accumulation of Aβ plaques	AChE	inhibit Aβ_1-42_ oligomerization, restore the cell viability	Shiao et al. (2017)
	*Caenorhabditis elegans*	inhibit the abnormal accumulation of Aβ plaques	—	improve the survival of CL4176 worms in response to proteotoxic-stress induced by Aβ protein aggregation	Chen et al. (2018)
	HEWL model system	inhibit the abnormal accumulation of Aβ plaques	—	antagonize amyloidosis, destroy the fibril structure, and convert amyloid fibrils into non-Shape aggregates, and inhibits the conversion of HEWL in a dose-dependent manner	Zhang et al. (2015)
	Acute hypobaric hypoxia C57 mice	anti-oxidative stress	HO-1, NQO1, γ-GCS, Nrf2	reduce HH-induced memory decline, increase the expression of nuclear factor E2 p45- related factor 2, heme oxygenase-1, NAD(P)H: quinone oxidoreductase 1, and γ-GCS synthetase in mRNA and protein levels	Zheng et al. (2019)
	*Caenorhabditis elegans*	anti-oxidative stress	DAF-16	trigger the nuclear localization of DAF-16. DAF-16 regulates target genes to participate in lifespan regulation and stress resistance	Mukhopadhyay et al. (2006)
	*Caenorhabditis elegans*	anti-oxidative stress	sod-3, hsp-16.2	extend the mean lifespan of worms and increase their survival under oxidative stress	Chen et al. (2018)
	Rats	neuroprotective effects	glutamate	reduce the 4-aminopyridine-evoked (4-AV) increase in cytoplasmic free Ca^2+^ concentration, decrease 4-AV-induced phosphorylation of protein kinase C	Lu et al. (2016)
	SH-SY5Y cells	inhibits apoptosis	TrkA/TrkB	inhibit the release of Cyt c and the activation of caspase-3 through the ERK pathway to achieve neuroprotection	Zhu et al. (2013)
	PC12 cells		Bax, Bcl-2	prevent a H_2_O_2_-induced increase of the Bax/Bcl-2 ratio, the formation of ROS, and accumulation of intracellular free Ca^2+^ ([Ca^2+^] i)	Kuang et al. (2009)
PD	6-OHDA subacute PD model mice	inhibits neuroinflammation	DA, DOPAC, HVA, NE, and 5-HT	the apoptosis of DA neurons is reduced, and the pathological state of PD is improved	Chen et al. (2007)
	MPTP model mice	inhibits neuroinflammation	DA, DOPAC, and HVA	prevent the level of DA, DOPAC, and HVA in the right striatum of MPTP model mice from decreasing	Zhang et al. (2021a)
	6-OHDA-induced PC12 cell model cell	inhibits neuroinflammation	DA	improve 6-OHDA-induced PC12 cell model cell viability, enhance redox activity and mitochondrial membrane potential, reduce ROS production, and inhibit mitochondrial-mediated apoptosis	Chen et al. (2019)
	SH-SY5Y cells	inhibits neuroinflammation	c5/c5a, IL-1β, IL-4, TNF-α, IL-2, p52, p38MAPK, NF-κB	attenuate DA neuron damage, regulate the activation of ROS/ATF3/CHOP pathway participate in the inhibition of inflammation in dopaminergic neurons, inhibits the occurrence of apoptosis and neuroprotective effect	Zhao et al. (2016), Zhang J et al. (2017), Liang et al. (2019)
	MPTP model mice	inhibits neuroinflammation	Iba-1	inhibit the small activation of glial cells improve inflammation in the brain	Ho, (2019)
	PD mice	inhibits neuroinflammation	NLRP3/CASP-1/IL-1β	improve the neuropathological state of PD mice through neuroprotective cell survival and inhibit activated microglia-mediated NLRP3/CASP-1/IL-1β pathway	Gao et al. (2020)
	MPTP subacute PD model mice	inhibits apoptosis	Bcl-2	inhibit the release of mitochondrial Cyt c and caspase-8 and the lysis of caspase-3 by reducing the ratio of Bax/Bcl-2 in dopamine neurons in the substantia nigra	Liu et al. (2019)
	SH-SY5Y cells	inhibits apoptosis	c5/c5a, IL-1β, IL-4, TNF-α, IL-2, p52, p38MAPK, NF-κB	inhibits the occurrence of apoptosis, and neuroprotective effect	Zhao et al. (2016)
	MPTP subacute PD model mice	regulates autophagy	p-AKT/AKT, P62	improve the neurobehavior of PD mice by upregulating the survival signal p-AKT/AKT, promoting the clearance of α-synuclein and the degradation of the autophagy substrate P62, exerting a neuroprotective effect	Zhang et al. (2021b)
	PD models	regulates autophagy	SIRT1, HSP70	activate FoxO1 to cause autophagy gene transcription, promote the autophagic degradation of α-synuclein, which can reverse the damage of dopaminergic neurons	Donmez and Outeiro (2013); Chen et al. (2019)
	MPTP model mice	regulates autophagy	GDNF	GFRα1 and TH in their brains, improve the pathological state of PD	Xu et al. (2016)
	6-OHDA-induced rat models	nourishment of nerves	BSCL2	reduce the activation of ERS related pathways	Zhang Y et al. (2017)
	MPTP model mice	improves mitochondrial dysfunction	mitochondria complex	reduce equivalents	Iverson (2013)
	SH-SY5Y cell line	improves mitochondrial dysfunction	mitochondria complex	attenuate cell damage and reverse complex Ⅰ by increasing the activity of complex Ⅱ to inhibit the induction of mitochondrial respiratory disorder and bioenergy weakness	Ma et al. (2019)
	6-OHDA-induced PC12 cells	improves mitochondrial dysfunction	—	The mitochondrial membrane potential was increased in 6-OHDA-induced PC12 cells and the state of mitochondrial energy disorder was improved	Ma et al. (2019)
ALS	neurons in SOD1 astrocyte conditioned medium	neuroprotective effects	GLT1	improve neuron survival and synapse loss treated with SOD1 astrocyte conditioned medium	Tian Y et al. (2021)
VD	VD model rats	neuroprotective effects	AChE	promote effect on the restoration of ACh and choline levels in the hippocampus and striatum of VD model rats, increase the activity of AChE	Liu et al. (2013)
	VD model rats	neuroprotective effects	GDNF	up-regulate the expression of GDNF in the hippocampus by regulating the level of mitochondrial oxidation, thereby reducing the ischemic damage	Liu et al. (2017)

## Glossary

5-HT: serotonin
6-OHDA: 6-hydroxydopamine
AD: Alzheimer’s disease
ALS: amyotrophic lateral sclerosis
APP: amyloid precursor protein
ARE: antioxidant response element
ATP: adenosine 5′-triphosphate
Aβ: Amyloid-β
BACE1: Beta site amyloid precursor protein cleaving enzyme 1
BDNF: brain-derived neurotrophic factor
BSCL2: Berardinelli-Seip congenital lipodystrophy type 2
Cyt c: cytochrome c
DA: dopamine
DOPAC: 3,4-dihydroxyphenylacetic acid
ECH: Echinacoside
eIF2α: eukaryotic initiation factor-2α
ER: endoplasmic reticulum
ERK: extracellular signal-regulated kinase
ERS: endoplasmic reticulum stress
ETC: electron transport chain
**FADH**_**2**_: flavin adenine dinucleotide
FoxO1: forkhead box subgroup O1
GDNF: glial cell-derived neurotrophic factor
GFRα1: GDNF family receptor α
GLT1: glutamate transporter 1
HD: Huntington’s disease
HEWL: hen egg-white lysozyme
HO-1: heme oxygenase-1
HVA: high vanillic acid
IIS: insulin/IGF signaling
IL-1β: Interleukin-1 beta
IL-2: Interleukin-2
IL-4: Interleukin-4
Keap1: Kelch-like ECH-associated protein-1
**MPP** ^**+**^: 1-methyl-4-phenylpyridinium
MPTP: 1-methyl-4-phenyl-1,2,3,6-tetrahydropyridine
MS: multiple sclerosis
mTOR: mechanistic target of rapamycin
**NAD** ^**+**^: nicotinamide adenine dinucleotide
NE: norepinephrine
NF-κB: nuclear factor-kappa B
NGF: nerve growth factor
NMDAR: N-methyl-D-aspartate receptor
NQO1: NAD(P)H quinone oxidoreductase 1
Nrf2: Nuclear factor-erythroid 2-related factor 2
OXPHOS: oxidative phosphorylation
p38MAPK: p38 mitogen Pro-activated protein kinase
PD: Parkinson’s disease
PERK: Protein kinase-like endoplasmic reticulum kinase
PhG: phenylethanoid glycoside
ROS: reactive oxygen species
SIRT1: sirtuin 1
SN: substantia nigra
SNpc: substantial nigra pars compacta
SOD1: superoxide dismutase 1
SRC: spare respiratory capacity
TH: tyrosine hydroxylase
TNF-α: tumor necrosis factor-alpha
VD: vascular dementia
γ-GCS: γ-glutamyl cysteine Synthetase
